# A novel *UBIAD1* mutation identified in a Chinese family with Schnyder crystalline corneal dystrophy

**Published:** 2009-07-29

**Authors:** Yang Jing, Chun Liu, Junmin Xu, Liya Wang

**Affiliations:** 1 Henan Key Laboratory of Keratopathy, Henan Eye Institute, Zhengzhou, PR China; 2 Department of Oncology, People’s Hospital of Dongguan, Dongguan, PR China

## Abstract

**Purpose:**

To identify the molecular defect causing Schnyder crystalline corneal dystrophy (SCCD) in a Chinese family with bilateral corneal abnormalities.

**Methods:**

The Chinese SCCD family was subjected to a complete ophthalmic examination that included slit-lamp examination and slit-lamp photography to assess and document the crystalline deposits and arcus lipoides. In vivo laser scanning confocal microscopy and Fourier-domain OCT were also performed on both eyes of SCCD patients. Blood samples were taken for subsequent genetic analysis. The two coding exons of the UbiA prenyltransferase domain-containing protein 1 (*UBIAD1*) gene were screened for mutations by direct sequencing.

**Results:**

We report on a novel heterozygous mutation of *UBIAD1*, G98S, in two patients with SCCD. The identified molecular defect cosegregates with the disease and is not found in 50 unaffected individuals. Morphological evaluation on SCCD by in vivo laser scanning confocal microscopy and Fourier-domain OCT highlighted pathological observations at the level of Bowman’s membrane and anterior stroma.

**Conclusion:**

The newly identified mutation expands the spectrum of mutations in *UBIAD1* that may cause pathological corneal cholesterol deposition. Observations by in vivo laser scanning confocal microscopy and Fourier-domain OCT were consistent with the previous histopathologic descriptions of SCCD.

## Introduction

Schnyder crystalline corneal dystrophy (SCCD; OMIM 121800) is an autosomal dominant disorder that results in clouding of the central cornea and premature development of peripheral arcus in the cornea. The clinical appearance of this dystrophy varies, but it is characterized by corneal stromal cholesterol deposition, most commonly in an axially distributed, annular or discoid pattern. The deposits are predominantly located in the anterior one-third of the stroma and Bowman’s membrane and are accompanied by a diffuse, gray stromal haze. Despite the name, Schnyder crystalline corneal dystrophy, only about half of the patients have detectable crystalline deposition in the cornea. The diagnosis of the disease in the absence of crystals is more challenging and has been reported to be delayed up to the fourth decade of life [1]. Progressive corneal clouding may be associated with loss of corneal sensation. Loss of corneal nerves has been demonstrated by confocal microscopy [2]. Eventually, phototherapeutic keratectomy or penetrating keratoplasty is required for recovery of corneal clarity and visual rehabilitation [3]. The dystrophy is considered rare, with less than 150 articles in the published literature, and most articles reporting only a few affected persons.

A genome-wide DNA linkage analysis in two large SCCD families of Swede-Finn descent established a 16 centimorgan (cM) interval between markers D1S2633 and D1S228 on chromosome 1p36 as the the chromosomal segment harboring the putative casual gene [4]. Theendakara et al. [5] further refined the SCCD locus using families of multiple ethnicities, reducing the candidate region to a 2.32 Mbp interval lying between genetic markers D1S1160 and D1S1635, or possibly a smaller 1.57 Mbp interval between D1S244 and D1S3153. Aldave et al. [6] and Yellore et al. [7] have reported sequencing of all annotated genes within the 2.32 Mbp interval, finding no pathogenic mutations and tentatively excluding them as causing SCCD, a finding proposed to result from locus heterogeneity, mutations within promoter or untranslated regions, the presence of an unannotated gene, or an error in the assignment of the candidate locus for SCCD due to misclassification of disease status in family members. Indeed, reanalysis of the pedigrees reported in an article by Theendakara et al*.* [5] showed a misclassification in one individual. The family which this individual belonged to had been used to define the centromeric boundary of the candidate interval at D1S1635. Weiss et al. [8] removed this family from the analysis and reevaluated the haplotypes in the other families. This resulted in a shift of the centromeric boundary of the candidate interval from D1S1635 to D1S2667. The expanded candidate interval included *C1orf127* (chromosome 1 open reading frame 127), *TARDBP* (TAR DNA binding protein), *MASP2* (mannan-binding lectin serine peptidase 2), *SRM* (spermidine synthase), *EXOSC10* (exosome component 10), *FRAP1* (FK506 binding protein 12-rapamycin associated protein 1), *ANGPTL7* (angiopoietin-like 7), *UBIAD1*, and *LOC39906*. Recently, Orr and coworkers [9] reported that mutations in the *UBIAD1* gene resulted in SCCD. *UBIAD1* spans 22 kb and the locus contains up to five exons with potentially several different transcripts. Up to date, mutations have only been described in exon1 and 2 which form a discrete transcript encoding a protein with a predicted prenyl transferase domain and up to eight transmembrane spanning regions.

Eleven different *UBIAD1* mutations causing SCCD have been reported to date [7-10], predominantly in Caucasian patients. Molecular genetic analysis of patients from diverse ethnic backgrounds showed both genetic and allelic heterogeneity for SCCD.

In the present study we report the molecular genetic analysis of *UBIAD1* in a Chinese family with SCCD, in which a novel *UBIAD1* mutation was identified. To our knowledge this is the first report of SCCD with novel gene mutation found in family from the mainland of China.

## Methods

This study was approved by the Institutional Review Board of the Henan Eye Institute. Informed consent in accord with the Declaration of Helsinki was obtained from the patients and their family members who participated in this study.

### Report of cases

#### SCCD Case 1

**Figure 1:**
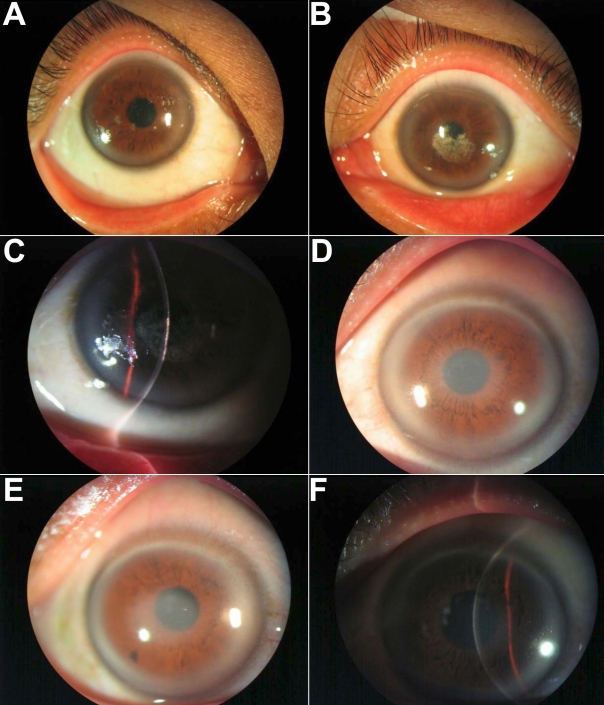
Slit-lamp photographs of affected patients eyes. **A**: The right cornea of the 29-year-old Chinese woman shows punctiform deposition of subepithelial crystals, central haze, and arcus lipoides. **B:** Appearance of her left cornea demonstrates a central plaque of subepithelial crystals slightly inferiorly displaced in the visual axis, midperipheral clouding, and arcus lipoides. **C:** Slit-lamp photograph of left eye (OS) demonstrating subepithelial crystalline deposits. **D:** The right cornea of the 54-year-old man shows central panstromal disc-like opacity obscuring the pupillary axis, and prominent arcus lipoids. **E, F:** Anterior stromal disciform opacity with arcus lipoides is observed in his left eye. No crystals are apparent in the corneal stroma.

A 29-year-old Chinese woman (the proband) was referred to our department in November, 2008 with symptoms of photophobia and vision decrease for about 1 year. Her best-corrected visual acuity (BCVA) in a lit room was 20/20 in the right eye and 20/100 in the left eye. Under slit-lamp examination, the right cornea presented with far less crystalline deposition than in left eye, but the central area showed mild anterior stromal haze. The left cornea showed typical central subepithelial crystalline deposits. The paracentral cornea was clear, but prominent arcus lipoids were present in both eyes. The deposits were composed of a fine, lacy garland of crystals present primarily in the anterior aspect of the stroma just beneath Bowman’s membrane (Figure 1A-C). Cochet-Bonnet esthesiometer was used to measure the corneal sensitivity and revealed no decrease of corneal sensation in both corneas. Intraocular pressures were 16 mmHg and the ocular examination was otherwise unremarkable.

Genu valgum has been postulated to be an independent trait reported in association with SCCD. Both the physical exam and radiography of knee joints demonstrated bilateral genu valgus in the patient. The blood serum level of total cholesterol was marginally elevated, as were levels of low-density lipoprotein, apolipoprotein A1, and apolipoprotein B. Triglycerides and high-density lipoprotein were normal.

#### SCCD Case 2

A 54-year-old man, the father of the proband, was seen in our department in April, 2009 with bilateral SCCD. He presented with similar symptoms as the proband. Best-corrected visual acuity was 10/20 in the right eye and 20/25 in the left eye. The patient didn’t seek medical attention until this point. Pupillary reaction, intraocular pressure, ocular motility, and fundus examination results were normal. Slit-lamp examination of both corneas demonstrated a mild central panstromal disc-like opacity obscuring the pupillary axis, and prominent arcus lipoids. These clinical changes were more prominent in the right eye. A clear midperipheral corneal stroma was noted between the central disc-like opacity and the arcus (Figure 1D-F). Corneal sensation was normal in both eyes. Fasting lipid profiles identified elevated serum cholesterol.

### Mutation Analysis

**Figure 2:**
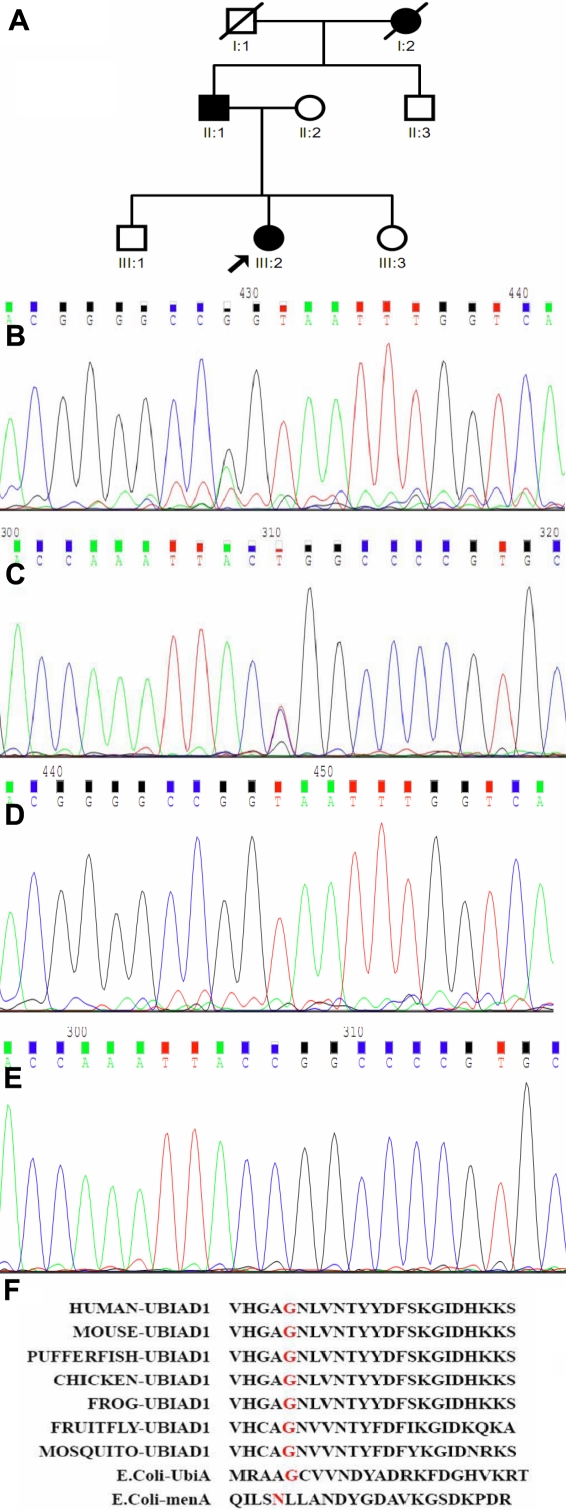
Novel *UBIAD1* mutation in the study family. **A**: Pedigree of the study family. In the family tree, squares indicate male and circles indicate female of the family members. Slash denote family member who was deceased, whereas heavy shading means the individual who was affected by SCCD. Arrow indicates proband. **B**: Sequence chromatogram of the proband and her father showing heterozygous mutation G98S in exon 1, forward reading. **C**: Reverse reading of the same mutation, showing G98S. **D**: Normal sequence of *UBIAD1* near codon 98, forward reading. **E:** Normal sequence of *UBIAD1* near the mutation in antisense strands detected in a healthy control. **F:** Sequence alignment of UBIAD1 around the amino acid residues of the mutation detected in the present study and in other species.

Molecular genetic analyses were performed in the two patients and unaffected family members: members II:2, III:1, and III:3 from this family (Figure 2A). Fifty healthy Chinese subjects were examined as controls. Genomic DNA was obtained from peripheral blood leukocytes according to standard procedures. For amplification of the exon1 and exon2 of *UBIAD1*; primer pairs were used as designed by Yellore et al. [7]. Their sequences are as follows: *UBIAD1*; F1/R1, 5’-CTC GTG GGG TGT AAG ACC CAC TT-3’/5’-GCG GCT TAA ATT AGA AAG CCA CCT-3’, *UBIAD1*; F2/R2, 5’-AGT GCC CAC CTG CAC AGT CTA AG-3’/5’-CAA ACT GGG CAG CTC CTT TAC AA-3’. The sizes of the amplified DNA fragments were 819 bp and 979 bp, respectively.

Each polymerase chain reaction (PCR ) was performed in a 50 μl reaction mixture consisting of genomic DNA (100 ng ), dNTP mixture (200μM ), 25μl 2X GC buffer I with MgCl_2_, forward and reverse primer (0.1μM each ) and 2.5 U Hotstar *Taq* polymerase (Qiagen, Hilden, Germany ). GC buffer was provided by Takara Biotechnology (Dalian) CO. Ltd. (Liaoning, China) and designed for amplification of templates having complex secondary structure or high GC content. Amplification reactions were performed under the following conditions: 4 min of denaturation at 95 °C followed by 30 cycles of denaturation at 94 °C for 30 s, annealing at 55 °C to 60 °C for 30 s, extension at 72 °C for 30 s, and a further extension step at 72 °C for 8 min. For direct sequencing, PCR products were purified using the QIAquick PCR purification kit (Qiagen), cycle sequenced with a Big Dye Terminator cycle sequencing kit (Applied Biosystems, Foster City, CA ) and directly sequenced on both strands using an automatic DNA sequencer (ABI Prism 377 Genetic Analyser; Applied Biosystems) according to the manufacturer’s instructions.

Nucleotide sequences were compared with the published cDNA sequence of *UBIAD1* (GenBank NM_013319.2) for each exon.

### Confocal microscopy and Fourier-domain OCT analysis

In this study, we coupled in vivo confocal microscopy and Fourier-domain optical coherence tomography (OCT) to evaluate the morphological changes of the cornea in patients with SCCD.

After a detailed explanation, a laser scanning in vivo confocal microscopy with a diode laser of 670 nm wavelength (HRT3/RostockCorneaModule; Heidelberg Engineering, Dossenheim, Germany) was performed on both eyes of the SCCD patients. Two-dimensional confocal images of the different corneal layers were acquired. Subsequently, the corneas of the proband were scanned three times with two scan patterns, the line scan and the cross-line scan, by a high-speed, high-resolution, Fourier-domain optical coherence tomography (Optovue Inc, Fremont, CA).We chose the best of the three scans for analysis.

## Results

### Mutation Analysis

Direct sequencing analysis of *UBIAD1* exon 1 in the proband and her father revealed a single heterozygous base pair transition at nucleotide position 292 (G→A), which converts a Glycine at codon 98 into a Serine, shown in Figure 2B. This mutation was confirmed by analysis of the reverse sequence data and has not previously been identified in SCCD (Figure 2C). The unaffected members of this family and normal controls didn’t have the mutation as determined by direct sequencing (Figure 2D,E). Therefore, the identified molecular defect cosegregates with the disease in this family.

### Confocal microscopy and Fourier-domain OCT Analysis

**Figure 3:**
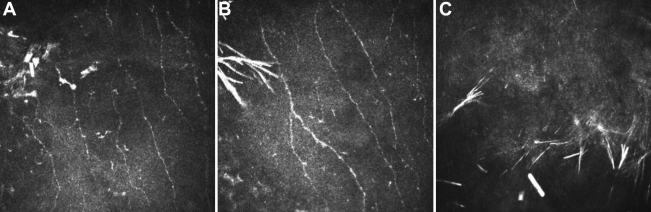
In vivo laser scanning confocal microscopic findings for the proband. **A, B:** Brightly reflective deposition, presumably cholesterol or lipid, is associated with the subepithelial nerve fibers in the left cornea. The shapes of the crystals were needle-shaped or rectangular. **C**: Anterior stroma showed the presence of multiple deposits of brightly reflective crystalline material. The keratocyte nuclei are undetectable.

**Figure 4:**
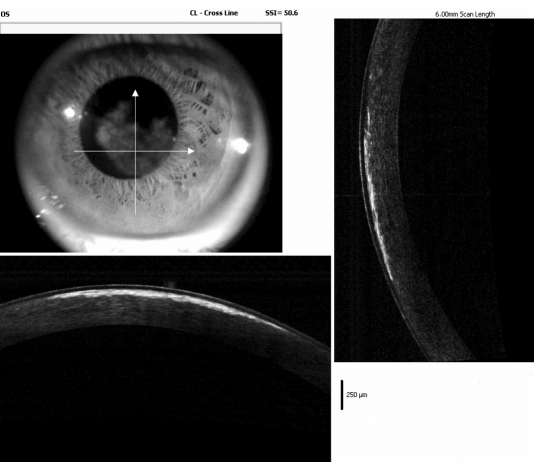
Fourier-domain OCT findings of the proband’s left cornea. Shown is the presence of crystalline deposits localized within the anterior stroma in OS. The highest density of crystalline deposits seemed to be mainly in the anterior stroma, within the first 100 µm of the corneal depth.

In vivo laser scanning confocal microscopy revealed pathological alterations of the normal corneal anatomy in the patients. Abnormal subepithelial nerve plexus were found in Bowman’s membrane, presenting with an irregular curved appearance and prominent beam-like structures. Large accumulations of brightly reflective crystalline material were noted in Bowman’s membrane. The anterior stroma showed the presence of multiple deposits of brightly reflective crystalline material extending from the anterior to the mid stroma. The shapes of the crystals were needle-shaped or rectangular (Figure 3A-C). However, the crystals were not apparent in both eyes of the father. The images acquired from Fourier-domain optical coherence tomography (OCT) indicated that the main presence of crystalline deposits was localized within the anterior stroma, extending from the basal epithelium layer to a depth of 100 to 150 µm (Figure 4).

## Discussion

SCCD is a rare autosomal dominant disorder that usually appears in the first or second decade of life. Up to date, only two patients with SCCD were reported from China (Taiwan), with heterozygous mutations (N102S, G177R). N102S is the most frequent mutation found in Caucasian SCCD patients with either European or unknown ethnicity [10]. Our patients are from a family in the central region of China, and the mutation is found to be novel. Moreover, our patients have no ethnic relationship with the Caucasian.

Of 28 unrelated SCCD-affected families with *UBIAD1* mutations reported previously, 11 different mutations have been reported (N102S, D112G, G117R, D118G, R119G, L121F, S171P, T175I, G186R, N232S, and D236E). The novel mutation identified in this study demonstrates further mutational heterogeneity. Studies of the genetic basis of the corneal `dystrophies have revealed that most reported cases of SCCD are caused by amino acid substitutions within *UBIAD1*, and most reported mutations have been missense mutations. The novel mutation identified in our current study is also a heterozygous missense mutation, and the site of the mutation is unique. The G98S mutation converts a Glycine at codon 98 into a Serine. The amino acid substitutions described as mutations in SCCD patients were examined for charge, size, and hydrophobicity to understand the consequences of these mutations on the UBIAD1 protein structure and function. Many of the mutations reported in prior studies were nonconservative amino acid substitutions. There were dramatic size and/or shape differences between the reference sequence and mutant amino acids in these mutations. Interestingly, the mutation locations identified in our study as well as prior publications revealed several clusters identified as loops 1, 2, or 3 when shown in a two-dimensional modeling [10]. Our novel mutation is located in loop 1, and this mutation seems to occur in parts of the protein located on one side of the membrane. Additionally, all mutations fall either in aqueous portions of the UBIAD1 protein or transmembrane helices close to one face of the lipid bilayer. However, it is unknown why mutation clusters are located on one side of the membrane. As shown in sequence alignment of the putative ligand/polyprenyldiphosphate binding site in loop 1 (Figure 2F), the mutation found in this study, G98, has been highly conserved among many species, indicating the importance of these proteins.

*UBIAD1* produces a protein that is predicted to contain a prenyltransferase domain and up to eight transmembrane spanning regions that could play a role in cholesterol metabolism. This gene is variably expressed in a wide variety of tissues, but its expression as measured by expressed sequence tag (EST) counts is higher in the eye than in any other tissue (UniGene’s EST Profile Viewer). Although *UBIAD1* has been found to be expressed in the adult human cornea (NEIBank library NbLib0073), the role of mutations in *UBIAD1* in the pathogenesis of SCCD is not well understood [7]. UBIAD1 can interact with the COOH-terminal portion of apoE [11], which is known to be involved in cholesterol transport, especially in mediating solubilization and in the removal of cholesterol from cells [12]. Hence, there is an obvious association between a defect in UBIAD1 and cholesterol transportation. However, UBIAD1 also contains the prenyltransferase domain, which is involved in cholesterol synthesis. Therefore, a defect in UBIAD1 may be associated with an increase in local cholesterol synthesis. The major weakness of this study is the small number of patients. Therefore, the potential consequences of the mutation described in this study on UBIAD1 protein function should be investigated in future study.

Though considerable phenotypic variation has been reported, the phenotype-genotype correlation is not clear. It still needs to be determined if the mutational pattern is correlated with the clinical course, or may be a prognostic indicator. As in our study, the proband and her father both had a G98S mutation, the 29-year-old proband had predominantly central ring pattern of crystalline deposition and her father had predominantly corneal opacity without crystals. The existence and nonexistence of crystals was confirmed by in vivo laser scanning confocal microscopy. A ring pattern of corneal crystalline deposition was noted in individuals of different ages and with different mutations. These findings demonstrate further the phenotypic heterogeneity and mutational heterogeneity in SCCD. It is possible that the phenotypic heterogeneity resulted from modulating influences such as environmental effects or that a specific phenotype may be a result of the interaction of multiple genes.

Recently, with the development of more sophisticated corneal imaging modalities, the microstructure of the living cornea can be observed in situ throughout the disease course, rather than only at the end-stage. The information gained from these techniques has enhanced our understanding of the pathophysiological mechanisms at play in the disparate disease entities. Confocal microscopy and Fourier-domain optical coherence tomography (OCT) are new emerging non-invasive technologies, which are useful as supplementary diagnostic tool for in vivo assessment of the histopathology of many ocular surface diseases and anterior-segment disorders. Our morphological evaluation on SCCD by in vivo laser scanning confocal microscopy and Fourier-domain OCT will not only clarify the pathological changes of this disease but serve to further clarify the clinical findings of this disease. In the presented cases, clinically diagnosed as SCCD, in vivo laser scanning confocal microscopy and Fourier-domain OCT highlighted observations at the level of Bowman’s membrane and anterior stroma, which is consistent with the result of the previous histological study of SCCD [13-15]. However, observations obtained by these two techniques are neither completely comparable with established clinical observations using the slit-lamp biomicroscope, nor with the structural descriptions of established ex vivo microscopical techniques. Therefore, clinical researchers will continue to report new descriptions, of this disease, using these two contemporary technologies.

In summary, our data add a novel mutation identified in a Chinese family with SCCD to the existing spectrum of *UBIAD1* gene mutations. Despite the rarity of this corneal dystrophy, the fact that affected Chinese family also has mutation in *UBIAD1* provides another evidence to support the hypothesis that SCCD is possibly caused by *UBIAD1* mutations.
